# How to eliminate pathogen without killing oneself? Immunometabolism of encapsulation and melanization in *Drosophila*

**DOI:** 10.3389/fimmu.2023.1330312

**Published:** 2023-12-06

**Authors:** Tomas Dolezal

**Affiliations:** Faculty of Science, University of South Bohemia, Ceske Budejovice, Czechia

**Keywords:** melanization, encapsulation, immunometabolism, parasitoid wasp, ROS, hemocyte, lamellocyte, phenoloxidase

## Abstract

Cellular encapsulation associated with melanization is a crucial component of the immune response in insects, particularly against larger pathogens. The infection of a *Drosophila* larva by parasitoid wasps, like *Leptopilina boulardi*, is the most extensively studied example. In this case, the encapsulation and melanization of the parasitoid embryo is linked to the activation of plasmatocytes that attach to the surface of the parasitoid. Additionally, the differentiation of lamellocytes that encapsulate the parasitoid, along with crystal cells, is accountable for the melanization process. Encapsulation and melanization lead to the production of toxic molecules that are concentrated in the capsule around the parasitoid and, at the same time, protect the host from this toxic immune response. Thus, cellular encapsulation and melanization represent primarily a metabolic process involving the metabolism of immune cell activation and differentiation, the production of toxic radicals, but also the production of melanin and antioxidants. As such, it has significant implications for host physiology and systemic metabolism. Proper regulation of metabolism within immune cells, as well as at the level of the entire organism, is therefore essential for an efficient immune response and also impacts the health and overall fitness of the organism that survives. The purpose of this “perspective” article is to map what we know about the metabolism of this type of immune response, place it in the context of possible implications for host physiology, and highlight open questions related to the metabolism of this important insect immune response.

## Introduction

Insects possess primarily innate immunity, which includes both humoral and cellular immunity. Antimicrobial peptides and the melanization cascade constitute humoral immunity, while hemocytes, which perform various immune functions such as phagocytosis, encapsulation, and melanization, provide cellular immunity. Thus, melanization, which occurs in both humoral and cellular responses, is an essential component of insect immunity, as we have learned especially from the work of Anthony Nappi (1, 2). Melanization is a process that involves the oxidation of tyrosine and the production of quinones, which polymerize to form melanin (3). The process has several roles, including a developmental role in cuticle pigmentation and the nervous system, an immune role in clot formation during wounding, and a role in phagocytosis, nodulation, or encapsulation of bacteria, fungi, and parasites (4). Cellular melanization is particularly utilized in the encapsulation of multicellular pathogens. I focus here on the infection of the *Drosophila* larva by a parasitoid wasp, which is the most extensively studied model (5).

An effective immune response requires appropriate changes in immune cell metabolism and associated changes in systemic metabolism (6–9). Although immunometabolism has been a popular topic in recent years, research on immunometabolism in insects has somewhat lagged (10). Very little is known about the metabolic changes associated with cellular encapsulation and melanization, which is primarily a metabolic response. The enormous nutrient requirements of this response have a major impact on the entire organism, and regulation of metabolism therefore affects not only the efficiency of the response, but also the development and physiology of the surviving organism (11). The production of toxic molecules is essential to eliminate the pathogen, but is harmful to the host (3, 12). Adjusting the strength of the response while protecting the host has significant evolutionary and ecological implications (13, 14). The pathogen interferes with the host’s immune response by targeting the metabolism of the response (15). A thorough understanding of all these relationships cannot be achieved without examining the complex metabolic changes that support this response. This article focuses on what is known about these changes and the open questions that remain.

**Figure 1:**
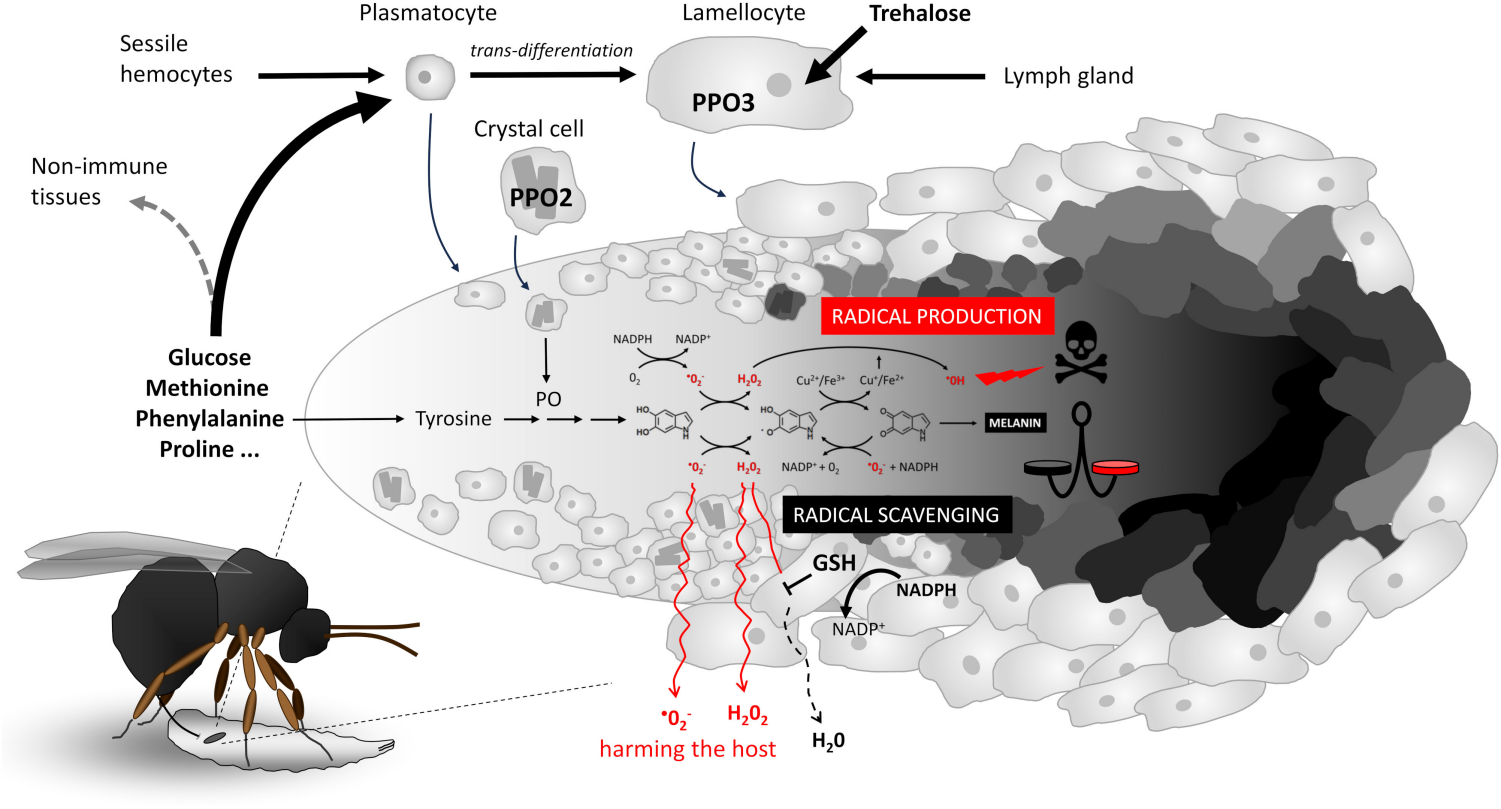
Cellular melanizing encapsulation response of *Drosophila* larvae to the egg of the parasitoid wasp *Leptopilina boulardi*. Schematic representation of hemocyte response, nutrient requirement, activation and differentiation, progressive adherence, encapsulation of the egg in multiple layers, and melanization (schematically shown from left to right on the egg). A simplified melanization reaction is shown in the middle, with the most critical redox interconversions between 5,6-dihydroxyindole, indole-semiquinone, and indole-quinone (shown by chemical formulas from left to right), the generation of reactive oxygen species to destroy the parasitoid (shown above the reaction), or the scavenging of radicals in cooperation with glutathione GSH to protect the host from the escape of radicals (shown below the reaction). The balance between radical generation and radical scavenging (indicated by the scale symbol) determines host resistance and protection. GSH, glutathione; PPO2/3, prophenoloxidase 2/3; PO, phenoloxidase.

During the reaction of the *Drosophila* larva to the parasitoid wasp egg (**Figure 1**), circulating plasmocytes are the first to recognize the parasitoid egg and activate immune response. This results in the mobilization of “sessile” hemocytes into the circulation (16), some of which attach to the egg, while others along with the originally circulating hemocytes trans-differentiate into lamellocytes (17). Once fully differentiated, the lamellocytes attach to the egg and encapsulate it in multiple layers. Additional lamellocytes are produced in the lymph gland. Encapsulation is associated with melanization, which is facilitated by the expression of prophenoloxidase PPO2 in crystal cells and PPO3 in lamellocytes (18). On the one hand, encapsulation and melanization ensure the elimination of the parasitoid by generating toxic molecules that are localized and concentrated on the egg inside the capsule, and on the other hand, they are presumed to protect the host by keeping this toxic reaction inside the capsule.

**Figure 2:**
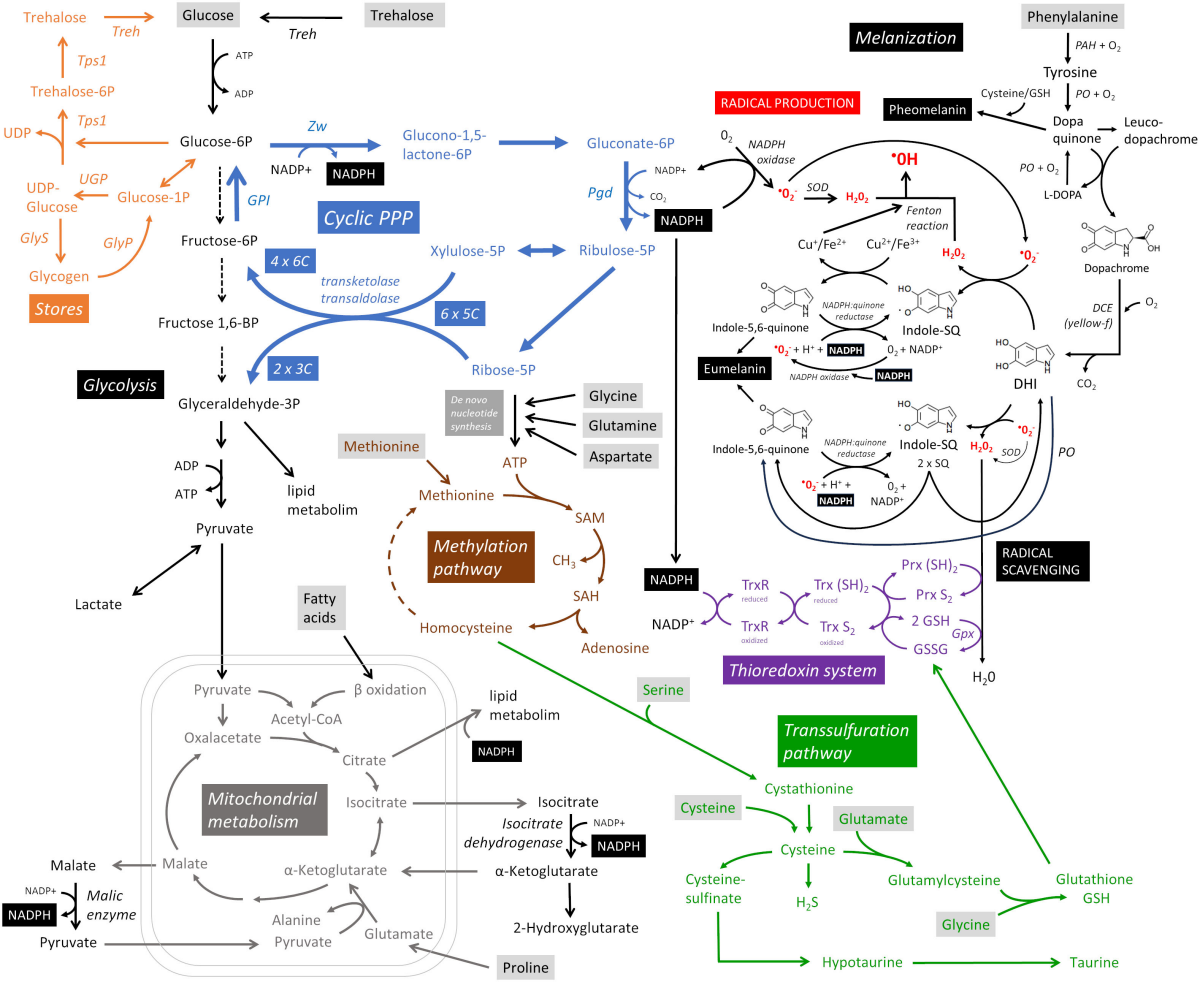
Metabolic pathways supporting cellular melanizing encapsulation. A detailed description is provided throughout the main text. Metabolites in gray boxes represent the predicted nutritional requirements of hemocytes during the response. Cyclic PPP, cyclic pentose phosphate pathway; DCE, dopachrome conversion enzyme; DHI, 5,6-dihydroxyindole; GlyP, glycogen phosphorylase; GlyS, glycogen synthase; GPI, glucose-6-phosphate isomerase; Gpx, glutathione peroxidase; Indole-SQ, Indole-semiquinone; GSH, glutathione; GSSG, glutathione disulfide; PAH, phenylalanine hydroxylase; Pgd, Phosphogluconate dehydrogenase; PO, phenoloxidase; Prx, thioredoxin peroxidase; SAM, S-adenosylmethionine; SOD, superoxide dismutase; Tps1, trehalose-6-phosphate synthase 1; Treh, trehalase; Trx, thioredoxin; TrxR, thioredoxin reductase; UGP, UDP-glucose pyrophosphorylase; Zw, Zwischenferment (glucose-6-phosphate dehydrogenase).

What are the metabolic requirements of hemocytes during parasitoid encapsulation and melanization (**Figure 2**)? Hemocytes maintain their basic vital functions in the resting state, i.e. without infection, and generate ATP with maximum efficiency from glucose by glycolysis and from fatty acids by beta-oxidation coupled to oxidative phosphorylation in mitochondria (19). This process allows them to produce up to 38 ATPs per molecule of glucose, although in reality it is lower. Once activated by the detection of a parasitoid, previously dormant processes are initiated in hemocytes. Unlike, for example, the activation of mammalian T lymphocytes, which require nutrient support for cell division, there is minimal hemocyte proliferation in this response (17, 19, 20). However, over a thousand genes undergo changes (9) that lead to intense transcription and translation, requiring nucleotide production and amino acid uptake to support them. During cellular differentiation, epigenetic modifications occur that require methylation. Methylation is also required for post-translational modification of new proteins. The methylation cycle intensifies during immune response, resulting in increased consumption of ATP and methionine, from which S-adenosylmethionine (SAM) is formed as a source of methyl groups (21). Differentiation of lamellocytes and encapsulation of the parasitoid by plasmatocytes and lamellocytes requires changes in the cytoskeleton and membrane, with cells adhering to the surface of the parasitoid where they interconnect to form layers of the capsule (22). Metabolically demanding is the production of reactive oxygen species (ROS) and other toxic molecules to destroy the parasitoid, but also the production of antioxidants to protect the host. Both actions are redox reactions that consume significant amounts of NADPH. NADPH is generated primarily by oxidative PPP, which uses glucose as its source (23), or by malic enzyme and isocitrate dehydrogenase, which require citric acid cycle intermediates (24). For melanization, the source is tyrosine, but this is more likely to be supplied to cells as phenylalanine and converted to tyrosine by phenylalanine hydroxylase (14). The role of mitochondrial function and ATP production in hemocytes during this immune response remains uncertain. However, lactate production is increased in hemocytes (9, 10), suggesting increased ATP production by glycolysis, which is much less efficient (2 ATP per glucose molecule) and consequently requires more glucose.

The primary purpose of cellular encapsulation and melanization is to produce toxic molecules that will eliminate the parasitoid, while localizing this production to the surface of the parasitoid and protecting the host (3). All processes are ultimately directed toward this goal. Both ROS and antioxidant production require NADPH. NADPH oxidase transfers electrons from NADPH to molecular oxygen, resulting in a one-electron reduction to the superoxide anion O_2_^-^. Of the three NADPH oxidases, most hemocyte types express *AIF*, but very little *Nox* or *Duox* genes (9, 19, 20). The conversion of O_2_^-^ to H_2_O_2_ occurs spontaneously or with the assistance of superoxide dismutase [the *SOD1* and *SOD2* genes are both strongly expressed in most hemocytes (9, 19, 20)]. These primary ROS (O_2_^-^ and H_2_O_2_) can be further processed to the much more reactive hydroxyl radical (^•^OH) or hypochlorous acid (HOCl) (3). The cyclic pentose phosphate pathway (PPP) is the most efficient mechanism for reducing NADP^+^ to NADPH, yielding up to six NADPH per glucose during the re-oxidation of glucose 6-phosphate (G6P) (23). In cyclic PPP, G6P is oxidized to phosphogluconolactone by glucose-6-phosphate dehydrogenase, reducing one NADP^+^. In the following step, 6-phosphogluconate dehydrogenase reduces another NADP^+^ while catalyzing the oxidation and decarboxylation of phosphogluconate to produce ribulose 5-phosphate pentose. The pentoses can be used for *de novo* nucleotide synthesis, but if more NADPH is needed, xylulose 5-phosphate and ribose 5-phosphate can be converted by transketolase and transaldolase to glyceraldehyde 3-phosphate (GAP) and fructose 6-phosphate. This hexose can be converted back to G6P by the reversed action of glucose-6-phosphate isomerase and oxidized again to produce more NADPH, forming a pentose cycle (cyclic PPP). Through the action of transketolases and transaldolases, six pentoses are recycled to four hexoses and two GAPs. Transketolases and transaldolases can also work in the opposite direction (25), using non-oxidative PPP to form pentoses from hexoses when the cell primarily needs to produce nucleotides.

*Drosophila* larval hemocytes metabolize glucose by cyclic PPP, and this metabolism is greatly increased during parasitoid infection (9) [detailed analysis of the expression of glycolytic and PPP enzymes in hemocytes is presented in (9)]. Another possibility for the formation of cytosolic NADPH is the utilization of the citric acid cycle intermediates, isocitrate and malate, which are transported from the mitochondria to the cytoplasm and metabolized by isocitrate dehydrogenase [*Idh* is strongly expressed in plasmatocytes, less in lamellocytes (9, 19, 20)] or malic enzyme [*Men* is strongly expressed in hemocytes, especially in crystal cells (9, 19, 20)] to alpha-ketoglutarate and pyruvate, respectively, with concomitant reduction of NADP^+^ to NADPH. The source in this case may be pyruvate from glycolysis, beta-oxidation of fatty acids, or proline (or possibly glutamine), which is converted in the mitochondria to glutamate and alpha-ketoglutarate, which may be converted in one direction to isocitrate and in the other to malate, thus serving as a source for NADPH formation. Proline is one of the most abundant amino acids in insect hemolymph (26–28), but it is not known whether it is used as an alternative source of NADPH in hemocytes during encapsulation/melanization. *P5cr* and *P5cr-2*, which are predicted to convert proline to pyrroline-5-carboxylate, and *P5CDh1*, which is predicted to convert L-glutamate 5-semialdehyde to glutamate, are expressed in hemocytes with stronger expression in lamellocytes (9, 19, 20). GAP formed by glycolysis or cyclic PPP, citrate and NADPH are also important for lipid metabolism, which is most likely essential during this reaction, at least for membrane remodeling during lamellocyte differentiation. All this can be provided by glucose metabolism and the coupling of cyclic PPP with downstream glycolysis and pyruvate metabolized in the citric acid cycle, or supplemented by proline metabolism in mitochondria. It is not known whether rewiring of mitochondrial metabolism, either for additional NADPH production by isocitrate dehydrogenase and malic enzyme or to support lipid metabolism, reduces ATP production by oxidative phosphorylation and thus the cell produces more ATP by glycolysis ending in lactate, whose increased production we detected during this response (9). The role of mitochondrial metabolism requires further investigation.

Melanization begins with the oxidation of tyrosine by phenol oxidase (PO) in the presence of O_2_ (29). Tyrosine is replenished in cells from phenylalanine by phenylalanine hydroxylase [*Hn* is expressed in hemocytes, more in plasmatocytes, less in lamellocytes (9, 19, 20)], so phenylalanine or tyrosine are essential sources for melanization. PO oxidizes tyrosine to dopaquinone, which is converted by intramolecular cyclization to leucodopachrome. The latter is oxidized to dopachrome by dopaquinone, while dopaquinone is reduced to L-DOPA, which is oxidized back to dopaquinone by PO. In an environment with cysteine or glutathione (GSH), cysteinyldopa and red pheomelanin can be formed (4). Whether this type of melanization plays a role in this immune response is unknown (30). Dopachrome is a substrate for the enzyme yellow-f (31), which decarboxylates it to 5,6-dihydroxyindole (DHI). *yellow-f* is one of the genes with the highest up-regulation in hemocytes after parasitoid challenge due to a strong expression in lamellocytes (9, 19, 20). In mammals, the tautomerization of dopachrome to DHICA (no decarboxylation), which is the basis of DHICA eumelanin, is also known; in insects, the formation of DHICA is unclear (4). The highly diffusible DHI and its redox conversion between DHI, indole-semiquinone and indolequinone probably represent the essential role that melanization plays in this type of immune response (3). Depending on the environment in which these redox conversions occur (pH, redox state, presence of antioxidants), this part of the melanization cascade can either greatly enhance the production of toxic radicals or, conversely, scavenge them. DHl is converted to indole-semiquinone in the presence of oxygen radicals to form H_2_O_2_. In addition to NADPH oxidase, the source of the oxygen radical may be the conversion of indole-semiquinone to indolequinone (3). Indole-semiquinone is further converted to indolequinone in the presence of Cu^2+^/Fe^3+^, reducing the ions to Cu^+^ and Fe^2+^, respectively. Cu^+^ and Fe^2+^ can convert H_2_O_2_ to a highly reactive hydroxyl radical (Fenton reaction), which locally damages various biomolecules at the site of its formation. When this reaction is directed to the surface of the parasitoid, it destroys it. Indolequinone can be reduced by NADPH:quinone reductase to indole-semiquinone (one-electron reduction) or DHI (two-electron reduction) (32), and the next round of oxidation could produce additional H_2_O_2_, ions, and ultimately ^•^OH. Unfortunately, it is not known whether NADPH:quinone reductase plays a role in melanization, so possible interconversion using NADPH is speculative.

However, in an environment with GSH, the same interconversions can scavenge radicals (3). DHI converts O_2_^-^ to H_2_O_2_, which is converted to H_2_O by GSH. Indolequinone, presumably involving NADPH:quinone reductase, scavenges O_2_^-^ to O_2_. In both cases, reactive indole-semiquinone is formed. The two indole-semiquinones can react with each other to form DHI (reduction) and indolequinone (oxidation), which can scavenge other O_2_^-^ radicals. Thus, in an environment where Cu and Fe ions are present, DHI-semiquinone-quinone interconversion can lead to radical production, whereas in an environment with GSH, it can scavenge radicals. Finally, indolequinone can polymerize into visible black eumelanin. DHI-semiquinone-quinone interconversions can occur spontaneously without enzymatic action. However, it is likely that enzymes (PO, NADPH:quinone reductase, NADPH oxidase) and the supply of Cu/Fe ions can direct these conversions in the desired direction. Whether NADPH is needed only for the input (NADPH oxidase) and for antioxidant formation (thioredoxin system), or whether it is also involved in melanization itself, is a question. In any case, the production and scavenging of radicals, the essence of this immune response, depends on sufficient NADPH production.

The parasitoid is destroyed by the production of radicals, and although their production appears to be tightly regulated and somehow localized and targeted to the parasitoid, the escaping radicals still threaten the host itself. Therefore, host protection by antioxidant production and ROS scavenging is an integral part of this immune response. We found increased levels of the reduced form of glutathione and taurine in hemocytes (9). Diptera lack glutathione reductase and therefore utilize a thioredoxin system in which the protein thioredoxin Trx S_2_ is reduced to the dithiol form Trx (SH)_2_ by NADPH-dependent thioredoxin reductase (TrxR) (33). Hemocytes strongly express *Trxr1* and *Trx-2*, many different peroxiredoxins and glutathione peroxidase *dj-1beta*, with increasing expression in lamellocytes (9, 19, 20). The reduced thioredoxin then reduces glutathione disulfide (GSSG) to GSH. GSH reduces H_2_O_2_ to water, which can also be reduced by thioredoxin peroxidase (Prx), which is also reduced by thioredoxin. Hemocytes express each component of this system very strongly, and lamellocytes appear to further increase expression, consistent with increased GSH production in hemocytes after infection. Thus, the thioredoxin system appears to be another major consumer of NADPH.

Increased glutathione and taurine must be newly produced in the hemocytes. The source is cysteine, which, if insufficient, is produced by the transsulfuration pathway from homocysteine, the source of which is methionine and the methylation pathway (SAM cycle) (34). Activated immune cells take up large amounts of methionine and significantly increase the methylation pathway (21). After ATP, SAM is one of the most abundant molecules in the cell. SAM is the source of the methyl group for the vast majority of methylation in the cell, both for the regulation of gene expression and for the methylation of newly generated biomolecules, especially proteins. In addition, the methylation pathway is the source of homocysteine for transsulfuration and the formation of GSH and taurine (34). The methylation pathway starts with the coupling of methionine and ATP, so its increase depends on the *de novo* production of ATP (the source is glucose and PPP) and the uptake of methionine. Thus, methylation and antioxidant production, which seems to be important for this immune response, are another reason for the need for glucose and amino acids such as methionine, glutamine, glycine, and serine in hemocytes.

Activation and differentiation of hemocytes takes place in the circulation, in sessile pockets or in the lymph gland, i.e. in environments with easy access to sugars and amino acids. However, melanization and related processes take place on the surface of the parasitoid and later in the capsule, which is formed by many layers of encapsulating cells that gradually die. In this environment, they probably have limited access to nutrients. Nevertheless, they must still metabolize intensively for several hours, certainly at least reducing NADP^+^ to NADPH. Therefore, it can be assumed that prior to encapsulation, hemocytes accumulate reserves of sugars and possibly other biomolecules (e.g., as a source of amino acids) for later use in NADPH production and melanization reactions. For example, mammalian neutrophils accumulate glycogen from which they then liberate glucose at their site of action, where sugars are no longer readily available (35). Insect hemocytes can potentially form glycogen or trehalose-6-phosphate stores (36) because they express the necessary enzymes (UGP, GlyS, Tps1) (9). They also express the enzymes required to release glucose from these stores (GlyP and Treh) (9), and the expression of these enzymes is markedly increased in differentiated lamellocytes (19, 20).

Although we have learned a lot about the melanization cascade through Nappi’s work, and more recently about supporting metabolism, especially cyclic PPP, there are still many unanswered questions:

1. How to achieve a localized toxic response that kills the pathogen but not the host? Do different types of hemocytes play different roles in this response? For example, are plasmatocytes and crystal cells primarily responsible for radical generation because they are the first to adhere to the parasitoid surface (37), whereas lamellocytes, which adhere later and form the outer layers of the capsule, are more likely to be responsible for protective functions and radical scavenging? If so, how are similar effects achieved in insect species that do not produce lamellocytes (5)?
2. Do Fe and Cu ions and Fenton reactions play an important role in melanization as suggested by Nappi? If so, how is their enrichment and controlled release on the pathogen surface achieved? Do Fe-transporting proteins such as transferrin (38, 39) play a role?
3. Do hemocytes store glycogen and trehalose, and is this essential for this response? Do they also store amino acids, e.g. in the form of storage proteins such as Lsp (40)?
4. Metabolism is very flexible. Does the metabolism of this reaction change based on the current nutritional status of the organism? Can proline or glutamine and NADPH production by isocitrate dehydrogenase and/or malic enzyme replace NADPH production by glucose metabolism via cyclic PPP?
5. How is the host protected against this reaction? Is there an essential role for the thioredoxin system in the production of antioxidants? Is the cysteine source or the methylation cycle and transsulfuration pathway essential?
6. How is the balance between producing and scavenging toxic radicals established? Too little production and/or too much scavenging will reduce resistance, the opposite will lead to more host damage. Thus, the balance is of great evolutionary importance.
7. All of these processes are potential targets for pathogen interference with the host immune response. How do they affect host-pathogen coevolution, and which ones become targets for perturbation by the pathogen?

## Data availability statement

Publicly available datasets were analyzed in this study. This data can be found here: https://www.flyrnai.org/scRNA/blood/; https://www.flyrnai.org/tools/single_cell/web.

## Author contributions

TD: Funding acquisition, Visualization, Writing – original draft, Writing – review & editing.
